# What Do Local People Really Need from a Place? Defining Local Place Qualities with Assessment of Users’ Perceptions

**DOI:** 10.3390/ijerph20021269

**Published:** 2023-01-10

**Authors:** Huiming Liu, Bin Li, Qing Liu, Yifan Li, Jing Zhao, Xuechun Wang, Chaoyi Cui, Shaoting Zeng

**Affiliations:** 1 Faculty of Humanities and Arts, Macau University of Science and Technology, Macau 999078, China; 2 Faculty of Innovative Design, City University of Macau, Macau 999078, China; 3 Laboratory for Urban Future, Peking University (Shenzhen), Shenzhen 518055, China; 4 Peking University Planning and Design Institute, Beijing 100087, China; 5 College of Art and Design, Beijing University of Technology, Beijing 100021, China; 6 School of Architecture and Built Environment, Queensland University of Technology, Brisbane, QLD 4001, Australia

**Keywords:** local place qualities, users’ perceptions, behavioural needs, using experiences, local cognitions, public space design

## Abstract

China is facing a serious urban regeneration issue in which replicable international-style locations are losing their socio-cultural adaptability, especially in anonymous residential neighbourhoods. This study defines the key location qualities from local literature and then refines these qualities through observation investigations and statistical analysis (*n* = 180) to establish links between theories and contemporary uses. Based on the results, a correlation analysis of local place qualities was assessed from users’ perceptions (*n* = 180) to identify the interactional influences between each indicator of local place qualities. Finally, local place qualities were scored to address their level of impact on users’ perceptions. The results highlighted health, enjoyment, and social dependence as the most concerning factors in site investigations based on local cognitions. They are strongly associated with key local place qualities (naturality, functionality, cosmological cognitions, and interdependent sociability), directly and indirectly resulting in different levels of impact on users’ perceptions at different scales.

## 1. Introduction

International architectural influences have created anonymous and placeless urban spaces through the process of globalization and rapid urbanization in Chinese cities over the past few decades [1,2,3]. Since the process of urban development does not effectively involve users’ participation [4,5], a lack of a clear definition of what constitutes a “good” place becomes a consequence that is addressed by the local perspective, and thus becomes one of the main issues in current urban regenerations [6,7,8,9]. Various scholars have emphasized the importance of local context and socio-cultural influences based on the theory of place, from place image [10,11,12] and place identity [13,14], to place attachment [15,16], and sense of place [17,18,19,20], which address the value and meanings the place holds for local people.

Many scholars have discussed objective and subjective aspects related to living conditions and how people perceive and evaluate these conditions from the perspective of liveability [21,22,23,24]. Lovell [25] considers a ‘liveable place’ as a ‘localised space’, which is a synthesis of the locally perceived and objective dimensions of a place. Place is a social space, which can be determined by its spatial implications for the social process, influencing the ways in which individuals and groups perceive, use, and shape their spaces [26,27,28]. It should be noted that the notions of places as social spaces open up the idea of a genius loci, which is a process in which people experience places beyond their physical or sensory properties and feel a sense of attachment to the spirit of a place [29]. Place is also determined as a socially defined space; individuals and groups perceive and attach meanings to their shared places because they are bound by a feeling of defined spaces [30]. It is the need for territoriality in which people can feel and define space by the function it serves, such as identity, security, and stimulation [23,31].

Studies from morphological and typological angles specifically examine how physical forms affect people’s urban experiences, adapt users’ behavioural needs, and support interactions [10,21,28,30,32,33,34,35]. On the other hand, environmental psychologists believe people’s bodily responses in urban spaces are physiological, behavioural, and cognitive reactions to the physical environment caused by positive stimulations of cognitions, emotions, and psychology [36,37,38,39,40,41]; locally inappropriate or unsupportive urban space may cause negative feelings and interactions with the physical setting that reduce the use of space, leading to mental stress, and even resulting in psychological problems or effects [37,39,42,43].

Current research using both physical and emotional factors to define place qualities is limited at the local level, because whether the qualities of places are capable of adapting to users’ needs, or fitting into the local context, depends on identification of the unique way of using the space under a specific socio-cultural effects [30,44,45,46]. However, the specific types of behaviours based on local cognitions are still missing from current research, especially in the context of China.

On the other hand, the place qualities defined in the existing global literature are better applied as a common guide in practice. Although they could enhance the general qualities of local places, determining the level of responsiveness and adaptability of local behavioural needs will require further studies, which encompass the unique forms of local behavioural needs, types of activities, and ways of interacting, alongside meanings and values, interpretations of local cognitions, and anthropological understandings of the worldviews beyond space design [28,47,48,49]. These may be found in studies of anthropologies, history, and sociologies, but how this knowledge both directly and indirectly affects people’s behavioural needs and perceptions of a place is still unknown, and this knowledge gap in the field of urban planning and design has yet to be resolved.

The importance of this issue is addressed not only in China, but also in many cities across developing countries in Africa and Asia [13,50,51,52,53], because the rapid urbanization process target economic growth, in which local values and meanings of socio-cultural needs are compromised for politico-economic opportunities. As a result, the urban built environment is presented in the form of high-raised modern and replicable buildings [7]. This is particularly evident in China’s development experiences.

**Figure 1:**
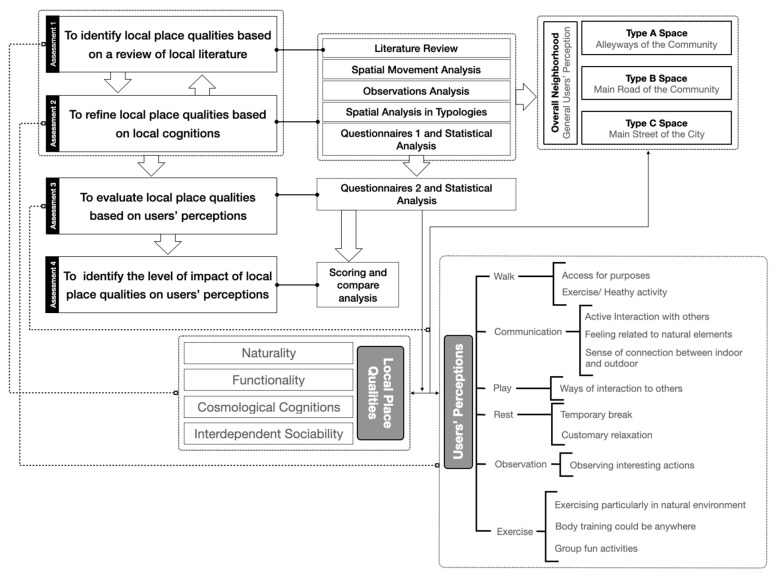
The research strategies of the study.

This paper, therefore, aims to define local place qualities that could improve the adaptation and responsiveness to local users’ behavioural needs, and the level of impact on users’ perception in the contemporary urban setting. Meanwhile, it also can be seen as an appropriate tool for evaluating the qualities of places from a local angle. To do so, firstly, identifications of local place qualities based on local cognitions, interpretations, and worldviews in the literature are employed. Then, users’ behaviours and activities are categorised to establish the links between practice and the local place qualities. Additionally, theoretical values and meanings are analysed via observations on site and interviews with users (*n* = 180). Finally, evaluating the place qualities based on users’ perceptions in different types of public spaces is crucial to define the aspects of local socio-cultural responsiveness to users’ perceptions and the qualities of their experiences (Figure 1).

## 2. Theory of Users’ Perception

Users’ perception in the urban design field is often addressed based on users’ values, preferences, and aesthetics [8,54,55,56,57]. The research of users’ perception not only includes studies of the physical, interpersonal, and cultural influences on the shaping of the built environment; it also represents the needs, actions, motives, and cognitive processes of users that build up requirements to reshape the local environment [8,54,57].

Perception is studied to determine how individuals sense the built environment and react to the understanding of stimuli from complex processing [57,58]. Ittelson [54] concludes that cognition, affection, interpretation, and evaluation of the built environment are the key dimensions needed for studying people’s perceptions based on their experiences of a place. To experience a place, individuals usually carry out three steps to build up the image and form a general picture of the place, starting by exploring local social relationships, cultural mores, and customs, to categorize the taxonomy of the physical setting in relation to other people’s actions and emotions, and finally systematizing the experienced built environment that related to individual’s own patterns of needs and activities [26,54,56].

However, people never passively experience their environment, as their actions are based on particular goals; thus, their explorations, categorizations, and systematizations of the physical setting are determined and limited by the qualities and conditions of a place [8]. Meanwhile, because of people’s past and present experiences through similarities in socialization, certain aspects of how people’s perceptions and expectations of the built environment will be held in common [59]. These common perceptions cannot only come from past experiences, but also from traditions, history, and cultural mores and customs that form unique local environments and special patterns of usage [60]. In turn, the actual forms, characteristics, and even qualities of a place somehow determine an individual’s needs and activities. These actions are changing in response to the changes in physical settings.

## 3. Identification of Local Place Qualities

Place qualities are also known as design qualities of public open spaces, which consider the design of a place with a broad range of positive outcomes in terms of economic, social, and health and environmental consequences [7,8,44,59,61,62,63].

In the Chinese region, the philosophy of harmony (balance) is the essence of urban transformation that is rooted in traditional philosophies, affecting governance and spatial constructions of cities, as well as individuals’ health and ways of living over time [64,65,66,67]. It explains the Chinese cosmological understanding of the interrelationship between humans and nature (and the cosmos) [65,68], and develops cosmological cognition to guide people to participate in natural progress for establishing individual lifestyles and improving bodily and mental health [7,65,66,67]. In addition, it also has a great impact on urban functions, which affect the spatial structure design and address the spatial connection between people and the physical setting [7,65,69,70]. Meanwhile, collectivism has been another key factor in Chinese urban development since 1949, creating a strong social tie and built-up interdependent sociability in social development, and it largely affects the urban forms and spatial structure of recent Chinese city design and planning [16,71,72]. Therefore, the importance of naturality, cosmological cognition, functionality, and interdependent sociability are highlighted as key place qualities, and further discussions will be carried out in the following sub-sections.

### 3.1. Naturality

Naturality does not solely refer to the phytological environment; it is also interpreted by various disciplines in China. The main idea of local cognition and perception of the built environment originated from Taoism, which believes humans and nature are cosmologically connected [66,67,73]. Taoism addresses nature as (1) the non-man-made environment [7], and (2) the flow of qi (energy of nature) [7,64]; while Confucianism understands nature as regulations and rules [69]; and Buddhism considers nature to be the symbolism of Heaven and Gods [74].

Despite the divine right and centralisation of political power in imperial times, nature is seen as mystical power or energy beyond phytological settings [68]. Firstly, from an environmental and geographical point of view, fengshui was understood to be the best tool to evaluate, recreate, and analyse the qi (energy of nature) of the physical environment for human habitation [65,68]. Qi, the vital cosmic current which runs through nature, can be scattered once it meets wind and can be stopped when it meets water [7,75]. By integrating greater amounts of qi, it can form shi (momentum or inclination), which is the essence and power of a place, and the inclination of the nature [70], referring to a position that has superiority and a latent dynamic force or power. This led to the formation of the interpretation of nature—which largely affects design aspects from a small scale, such as individual buildings, to city-scale changes—which is based on the movement of qi (energy of nature) and shi (momentum or inclination) as the borrowed power of nature that achieves harmony between human life and nature.

### 3.2. Cosmological Cognition

From an individual point of view, cosmological cognition is another key to understanding the philosophy of harmony. Chinese people believe that nature and humans are an indivisible body, with humans acting as a part of the natural procedure that must have a direct connection to nature not only for physical interactions but also for the spiritual system of individuals [67]. This addresses the cosmological cognitions of harmony that form the relationship of integration and connection between the individual and nature, particularly in activities and behaviours undertaken in public spaces, such as morning exercise, which was emphasized by Chinese medicine [67,68].

### 3.3. Functionality

Urban functions such as accessibility and a sense of connectivity are also important at different scales of city design. On one hand, by addressing the central location of the space, ancient Chinese astrology established the connection between the imperial power of the heavens, called the divine right [68]. The idea of a central point was then applied at various spatial scales throughout the design of buildings, blocks, and cities, to emphasize the social hierarchy and build up the rules for the governance [68,69,70]. Examples of this include the following:

(1) Beijing has designed the palace within the centre of the imperial city, and the imperial city within the centre of Beijing [65,70];
(2) The eldest lives in the centre of a courtyard house [69].

While on the other hand, based on the theory of canopy-heaven [7,74], the forms of the cities, as well as blocks and buildings, were designed in a square shape to symbolize stability. This is because ancient Chinese people believed the earth was square and the sky enveloping the earth was round [7]. Thus, in understanding city as the essential component for human habitation on the earth, structures ranging from city layout to building structure were all designed to imitate the square shape of the earth as a symbol of balance [7]. This creates great accessibility and spatial order in the street network. However, due to the growth of the urban population since the 1970s, rapidly increasing housing demands led to a large amount of uncontrolled self-regeneration in existing houses across China [7]. The spatial structure from the city level down to the neighbourhood level has dramatically changed, and consequently, the accessibility and the sense of connectivity are particularly changed at the neighbourhood scale.

### 3.4. Interdependent Sociability

Although the interpretation of nature and cosmological cognition play significant roles in Chinese urban development and the formation of unique behaviours, the transition of social ideologies also has a great impact on change in urban transformation. In 1949, collectivism created a special social phenomenon, which is what Spira called ‘qun-sim’ [76], and he defines qun-sim as having positive connections in the sense of interdependent social relationships [77], where individuals rely on each other to form a social group. This social phenomenon is seen as an important social constitution in Chinese society, in which collectivism led people to adapt to a collective lifestyle [78]. They have to rely on each other to fit into society, not only in terms of working relationships, but also in sharing life and living spaces [16,71,72]. This impact has changed the ways in which spaces are used, particularly in terms of group activities in urban spaces, such as group dance in Chinese cities.

## 4. A Complex-Developed Neighbourhood—Qian Yuan’en Si

**Figure 2:**
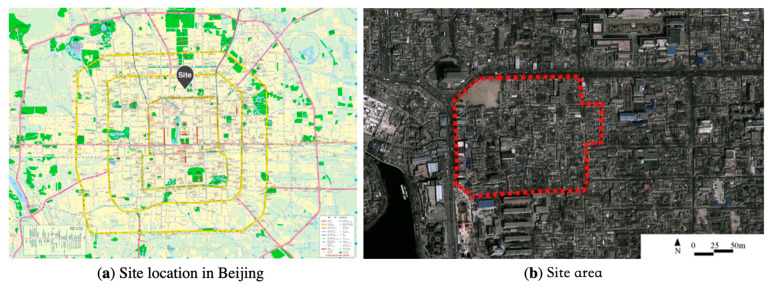
The location of Qian Yuan’en Si neighborhood in Beijing.

**Figure 3:**
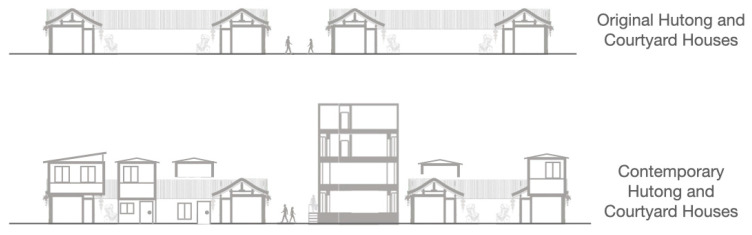
The section of Qian Yuan’en Si neighborhood.

Qian Yuan’en Si Neighborhood is a diverse neighbourhood located in the historical area of central Beijing (Figure 2), where mainly vernacular but also a variety of other building typologies were freely planned after 1980 [9,79,80]. The area is a typical result of conflict development between global impacts and changing local needs in Beijing, which present the transforming urban development strategies between formal urban planning and self-regenerated housing from the very early stage to contemporary development in China [7]. During the urban transformation, the main driver of changes in socio-spatial patterns was the shift in the formation of the economy from a politically controlled to a market-driven mechanism [7,9,72,81,82]. Although horizontal, the original fabric can still be recognized in the current urban tissue; vertically, the building facades and high-rise plots show dramatic changes [9] (Figure 3).

The reasons for choosing Qian Yuan’en Si neighbourhood as a case to study are (1) the latest development is largely influenced by contemporary modern design, which is hard to define using patterns linked to local cognitions. In order to identify how local design philosophy and principles are applied in the physical design that affects people’s using behaviours and perceptions, a historical-based spatial setting with multi-typological modern buildings provides an opportunity area to study and identify local location qualities, and (2) how these spatial qualities affect using experiences, and in what ways. Meanwhile, the neighbourhood contributes to the overall urban analysis because its built form combines a variety of building types in a vernacular context, which creates diverse and rich user experiences (Table 1). These are important to identify how the vernacular-built environment is designed based on the local cognitions that generate local place qualities, and then influence users’ behaviours and perceptions.

The research is designed in two stages to identify how local place qualities are evaluated by users’ perceptions based on local cognitions in the uses of public spaces, and how they can be distinguished from place qualities defined in global theories. The two stages are (1) to establish theoretical links between investigated behavioural needs from the case study and identified local place qualities in theories, and (2) to revise local place qualities based on investigations of users’ perceptions in the case-study neighbourhood.

## 5. Establishment of Theoretical Links between Users’ Behaviours and Local Place Qualities

Reviewing key literature allows us to identify the core theories that affect the public space design and adaptation of local needs in Chinese cities and to verify the actual behavioural needs and uses in practice could effectively justify these theories [83,84]. In order to do so, site observation is used in this study to identify how people use public spaces and for what types of activities, behaviours, and interactions the spaces are used for [62,85]. Then, by mapping their spatial movements at different times during the day, we define popular spaces in use [10,86,87,88]. Finally, we interview people about their reasons for choosing a particular time to use the space for certain activities to establish the theoretical links for refining local place qualities.

### 5.1. Investigation of Local Behavioural Needs

**Figure 4:**
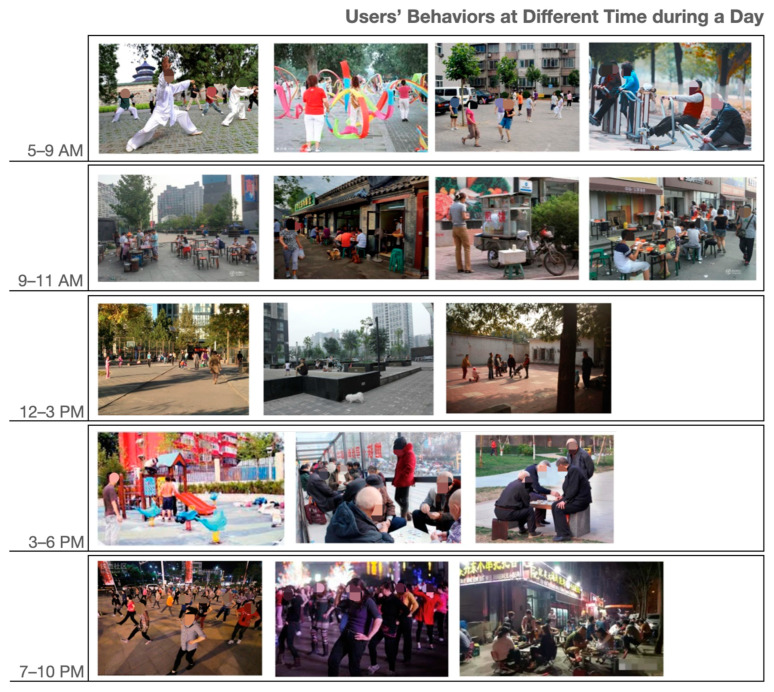
Users’ behaviours at different times of day.

**Figure 5:**
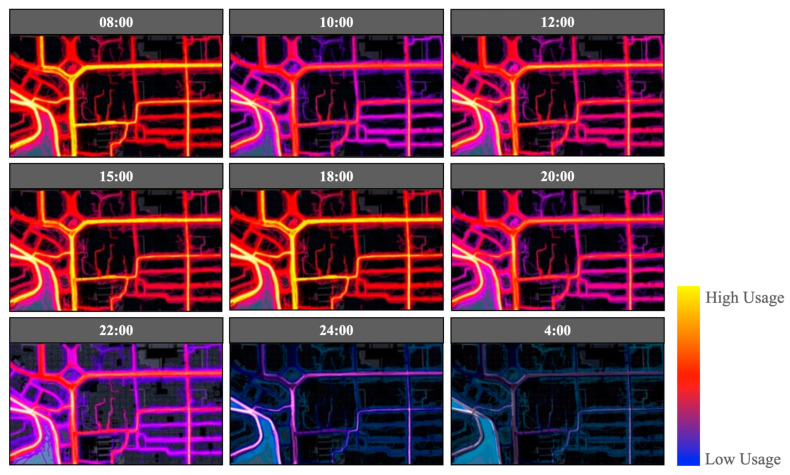
Pedestrian movement at different times of day.

**Figure 6:**
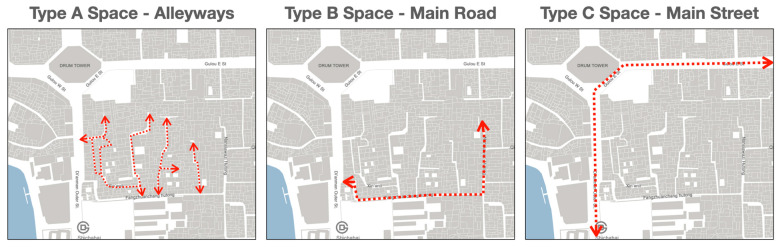
Three types of spaces identified in the neighbourhood.

The investigation was carried out on-site through the day, in an attempt to record all types of behaviours in public spaces at different times. Based on on-site observations, users’ daily behaviours can generally be divided into five categories, which are early morning exercise (usually 5 a.m. to 9 a.m.), morning essentials (usually 9 a.m. to 11 a.m.), noon activities (usually 12 a.m. to 3 p.m.), afternoon recreations (usually 3 p.m. to 6 p.m.), and nightlife (usually 7 p.m. to 10 p.m.) (Figure 4). Meanwhile, the spatial analysis of pedestrian movement data from STRAVA database (Figure 5) and on-site observation address three types of high-usage spaces, which are alleyways (Type A Space), main roads within a neighbourhood (Type B Space), and the main street around the neighbourhood (Type C Space) as shown in Figure 6.

### 5.2. Local Interpretations of Public Behaviours in Public Space

Based on categories of time in the uses of different types of public spaces (Figure 5 and Figure 6), 180 users were randomly selected on-site and interviewed to investigate how local users interpret their behavioural needs in public spaces (the statistical results are shown in Table 2). The selection of the random users was carried out in the public spaces of the selected neighbourhood for over 3 months. Over 60 percent of households in the neighbourhood were involved. Females showed more active participation in the investigation than males in the neighbourhood. On the other hand, as an aged society, retired people are the main user group in public spaces during the daytime, and this explains why people over 50 years old were the major user group in selected case study area (55 years old is the official age for female retirement in China).

**Figure 7:**
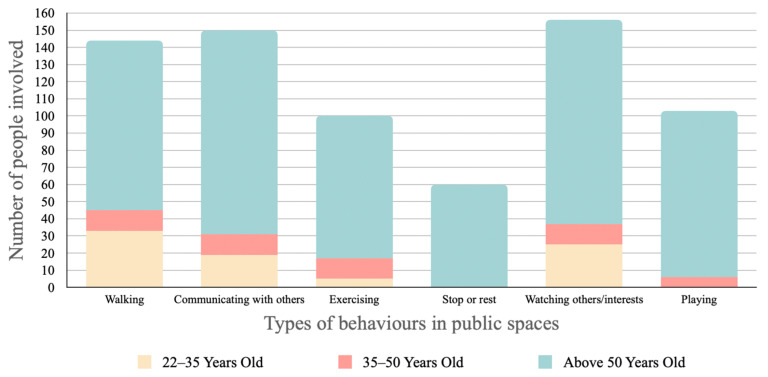
Local cognitions-based behaviours in public space of the neighbourhood.

**Figure 8:**
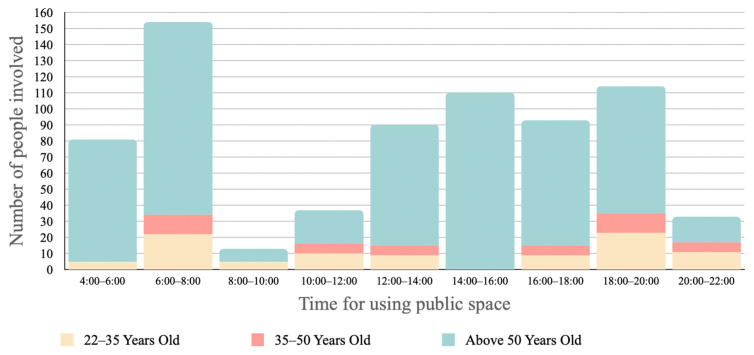
Time slots for different age groups in the use of public spaces.

Six types of behaviours commonly addressed in the use of public space from site investigations and interviews with local residents include walking, communication, play, rest, watching, and exercise, which encompass a range of uses, from fulfilment of psychological needs to socio-cultural uses (statistical results shown in Figure 7). However, as elders are the majority group in the neighbourhood who spend a large amount of time using public spaces, the study thus takes further steps to look at these users’ behaviours in detail every two hours compared to the general categories, to define the daily behaviour trends (statistical results shown in Figure 8). From the statistical data, very limited numbers of young and middle-aged people stop to interact with others compared to elders. Their routine is more focused on basic functions with fewer interactions, and they mostly use the public space in the morning and evening. On the other hand, elders are relatively active in their use of public space, interacting with others, comfortably walking around, and enjoying visual interests throughout the day.

In order to identify different age groups’ specific reasons for using the public space based on local cognitions, firstly, the interview questions were designed based on global common theories to identify why people walk, communicate, exercise, take breaks, watch, and play in public open spaces [8,89,90,91,92]. Then, how local people behave with local cognitions was studied. The analysis of reasons for using the public space (Table 3) shows the following:

(1) Users prefer walking for health reasons rather than walking to reach a specific destination. This was especially true of elders;
(2) Users present social connection and dependence as important aspects of communication. This was true for most of the participants;
(3) Elders believe exercising with other people is important;
(4) Users enjoy sunshine and fresh air as important aspects of public space;
(5) Socially interesting activities are significant when it comes to attracting people to watch in a public space;
(6) Most elders play in the public space because of the social attraction of activities.

Therefore, health, enjoyment, and social dependence can be seen as the most significant factors that affect the use of public spaces.

Although users’ behaviours are analysed through interview data, local cognitions are also key to defining whether they play a significant role that lead people to act out certain behaviours in public spaces. Thus, by reviewing further local literature that particularly focuses on these behaviours, the understandings can be summarized as:

#### 5.2.1. Walking

Within the ideology of Chinese philosophy, the term ‘walk’ has two levels of meaning: (1) access purposes, which means passing through from location a to location b [8,35,45,93]; (2) a health behaviour, which means walking with no particular destination in mind, but as a kind of healthy exercise that mainly happens after meals to digest food or to gather fresh qi (natural energy, refer to Section 3) [67,68]. Traditional Chinese medical science particularly addresses the spleen and stomach as belonging to the earth, which requires constant nurturing and ploughing for the soil to stay fertile for growing agricultural produce, preventing it from growing barren. Similarly, it is essential to regularly exercise the body and free the mind to maintain a healthy state [66,67].

#### 5.2.2. Communication

There are three ways to understand ‘communication’ in the Chinese context, which are as follows: (a) Active interaction with others. This refers to the interaction between people, such as in the early socialist time when courtyard houses became ‘chaotic yard’ houses under the influence of collectivism and socialism, in which indoor spaces were limited and external public spaces became an interactive platform that extended people’s lives, which helped form a special relationship between people [78,94,95]; (b) Feeling related to natural elements. This was explained in the theory of Taoism, which states that nature exists beyond physical boundaries such as buildings, encouraging people to engage with external space as a kind of communication with nature [67,70]; (c) Sense of connection (private to public), which refers to a strong sense of connection between the inside and outside of buildings that determine people’s level of willingness and the frequency with which they use public spaces [7,64,80].

#### 5.2.3. Play

Play refers to the ways of interacting with others, forming strong social connections and dependence. Concerning social interaction, in Chinese society, there is a strong social orientation based on the formation of local behaviours and social networking, which can be defined as ‘gregariousness’ or collectivism [76,77,94]. Based on this theory, Chinese people need to live within a social niche within a broader society. The social niche provides a sense of company, and without it, people become isolated and disconnected from the broader social network [77,78,94,95]. Therefore, ‘play’ is a way of engaging with people, which allows people to enter a social niche, have a social life or find a sense of social belonging.

#### 5.2.4. Rest

The resting behaviours in public spaces can be understood as (1) a temporary break in the public space [8,33,96]; (2) customary relaxation, which is either commonly seen as an engagement and relaxation within a natural setting based on local Taoism ideology [7], or enjoying a social action while surrounded by people [97].

#### 5.2.5. Observation

Observing interesting action is the most popular behaviour identified in the investigation. The action of observation is understood as visual interest from place theory, which is one of the keys to determining the success of the place [8,10,44], but it is understood slightly differently in China. Affected by collectivist experiences and Taoism ideology, the social connection is the main driver that forms social cohesion in the community to attract local users, who either participate in a social activity or watch a social activity [7,77]. In the site investigation, almost all elder users enjoyed observing interested actions while chatting with neighbours. Despite their limited mobility, observation satisfies their social and psychological needs within the community.

#### 5.2.6. Exercise

Based on the Taoism ideology and Chinese medicine, the best exercise time is between 5 a.m. and 7 a.m., as this is the ideal time to efficiently engage with qi (energy of nature) [66,67]. For this reason, the time slot between 6 a.m. and 8 a.m. is when daily use of public spaces is at its peak (Figure 4 and Figure 5). Through the site investigation, three types of exercise were identified: (1) exercising in the natural environment, which gathers the best amount of qi (energy of nature) from nature to benefit mental and physical health [67]; (2) body training, which can take place anywhere, depending on the availability of space; (3) group fun activities, which address the significance of social dependence and connections.

### 5.3. Labelling and Linking Users’ Behaviours to Identified Local Place Qualities

The analysis of understandings of public space from local perspectives shows clear theoretical connections with the identified local place qualities, from basic uses and behaviours in public spaces to psychological needs, which are met physically and visually in interactions with other people and the built environment. The theoretical connections are presented below in Table 4 and Table 5.

Based on the theoretical links between local place qualities and users’ behavioural needs on-site, a questionnaire is designed to identify local perceptions in relation to the use of public spaces in this complex-developed neighbourhood (Table 6).

## 6. Revising Local Place Qualities Based on Users’ Perception

**Figure 9:**
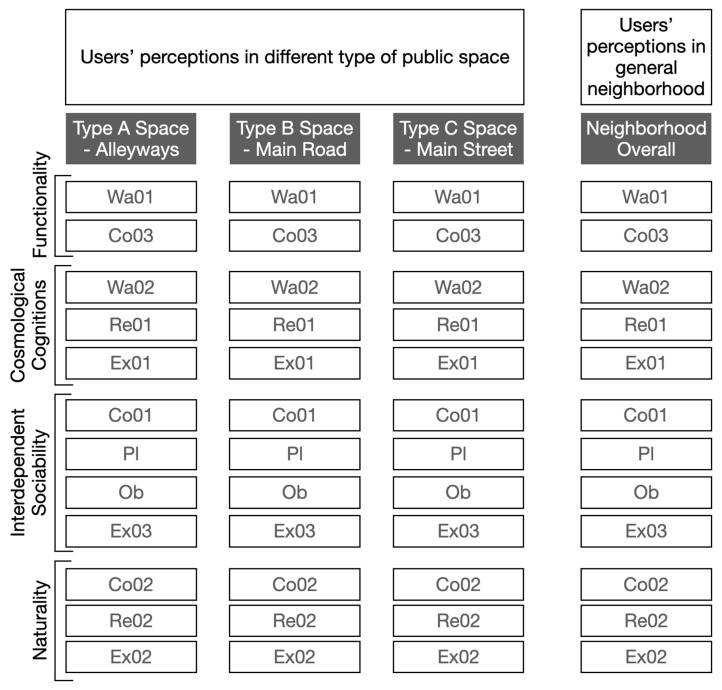
The structure of analysis between the type of spaces and local place qualities.

As discussed previously, each local place quality is highly related to others, and thus, in order to define the level of importance of each local place quality that affects users’ perceptions and perceived behavioural needs, Pearson correlations are employed to define the level of relevance between indicators and their belonged place qualities (Figure 9). This is important because the identification of the significance of aspects that affect users’ behaviours provides clues or guidance for future regenerations or developments that create locally responsive and culturally appropriate public spaces, especially in adaptations of local behavioural needs.

Based on the questionnaire developed from the last section (Table 6), the same interviewees (*n* = 180) who participated in the front part of the investigation were consulted. A five-point Likert scale was applied to qualify their perceptions [20,98]. Twelve indicators were evaluated in each type of public space (Section 5.1, Figure 4), and were followed by the analysis of the overall neighbourhood (Figure 9). The interview questions were designed to obtain four datasets (Table 6), which were used to evaluate the users’ perceptions of local place qualities.

The data collected from the interviews were processed in two steps. Firstly, reliability and validity tests were conducted, and then we performed a correlation analysis of Space A, B, C, and the overall dataset on twelve indicators in SPSS Version 26, in order to define how the indicators interact and affect users’ perceptions:

(1) $$\rho =\frac{cov\left(X_{ij}Y_{ij}\right)}{\sigma X_{i}\sigma Y_{i}}=\frac{E\left(\left(X_{i}-\mu x_{i}\right)\left(Y_{i}-\mu y_{i}\right)\right)}{\sigma X_{i}\sigma Y_{i}}$$

(2) $$i_{1,2,3,4}=\left(Function,\;Cosmology,\;Interdependent\;Sociability,\;Naturality\right)$$

(3) $$j_{1,2,3\ldots .,12}=\left(Wa01,Wa02,Co03,\ldots ,Ex02\right)$$

The correlation scores (r) were evaluated according to Cohen (2013) Cohen [99]’s standard in three main categories: small = 0.10; medium = 0.30; and large = 0.50.

Secondly, based on the correlation analysis (large and medium), the applications of the formula below are employed to identify the level of impact of each indicator of local place qualities on users’ perceptions, allowing us to define the level of significance of the local place qualities in relation to users’ perceptions.

(4) $$I_{j}=\overset{m_{a}}{\underset{l_{b}}{\sum }}j$$

The Impact Score (*I*) is calculated based on number of influences to the indicator of *j*; m presents the number of medium relevance from other indicators, *a* = 0.5, and *l* is a number of particular relevance compared to other indicators, *b* = 1.

### 6.1. Descriptive Statistics

The internal consistency of the scales is tested for the four main datasets and the individual scales related to each indicator. The Cronbach’s alpha values of 0.6–0.7 are the lowest acceptable threshold used in exploratory research [100]. The items in each dataset of type of space have met the threshold and showed internal consistency, as shown in Table 7 and Table 8. According to Kline [101], each indicator in each dataset has met the criteria of the normality test for acceptable skewness (values between +3 and −3) and acceptable kurtosis (values between +8 and −8). All data were tested for validity, and the significant correlations of each indicator were shown to be valid and reliable (results shown in Table 7 and Table 8).

Validity tests have been concluded by using Pearson correlations in SPSS, where the significant correlations of each item with the total score indicate the validity of those items (results shown in Table 9).

### 6.2. Analysis Results of Users’ Perceptions in Relation to Local Place Qualities in Different Types of Spaces

The correlations between each indicator were calculated at the three types of spaces and the overall neighbourhood, in which large correlations were highlighted to address the importance of relevance. In Type A Space—the alleyways of the community (Table 10)—naturality has been addressed its significance with its indicators of Co02, Re02, and Ex02. The corrections between them are r = 0.639 and r = 0.504. Then, naturality presents its large impacts on Ob at interdependent sociability (r = 0.649), and Co03 at cosmological cognitions (r = 0.752). Finally, Wa01 links its influence from functions to Re01 at cosmological cognitions (r = 0.509).

In Type B Space—the main roads of the community (Table 10)—the indicators of naturality have been highlighted as the largest impacts between each other, with Co02-Re02 = 0.725, Co02-Ex02 r = 0.594, and Re02-Ex02 r = 0.732. On the other hand, naturality has a significant influence on Co03 at functions: Co02-Co03 r = 0.683 and Ex02-Co03 r = 0.520, and to Ob at interdependent sociability, so that Co02-Ob r = 0.537. Meanwhile, cosmological cognitions bridge impact to functions as Re01-Wa01 r = 0.506.

In Type C Space—the main streets of the community (Table 10)—cosmological cognitions play an important role, influencing interdependent sociability (Re01-Pl r = 0.554 and Ex01-Co01 r = 0.5000) and functions (Re01-Wa01 r = 0.634). In addition, indicators of naturality show strong correlations as Re02-Ex02 r = 0.571; meanwhile, naturality also presents its influence on interdependent sociability (Co02-Ob r = 0.636) and functions (Co02-Co03 r = 0.602). The indicators of functions address their interactions as Co03-Wa01 r = 0.648.

In the perception of the overall neighbourhood (Table 11), naturality continues to present its significance not only between indicators (Co02-Re02 r = 0.639 and Re02-Ex02 r = 0.614), but also in terms of its large impact on interdependent sociability (Co02-Ob r = 0.610) and functions (Co02-Co03 r = 0.686). Cosmological cognitions present the general impact on functions (Re01-Wa01 r = 0.552).

Based on these key results of the analysis, the key findings will be discussed in the following section.

### 6.3. Key Findings

Based on the analysis above, the key findings of the research are twofold: Firstly, identifications of significant correlations between behavioural needs in relation to local place qualities are addressed, which explore how indicators of each local place quality interact with others that affect users’ perception. Secondly, scoring each indicator of local place quality based on the correlations allows us to define the level of impact of local place quality on users’ experience.

#### 6.3.1. Identifications of Key Indicators of Local Place Quality Affect Users’ Perception

**Figure 10:**
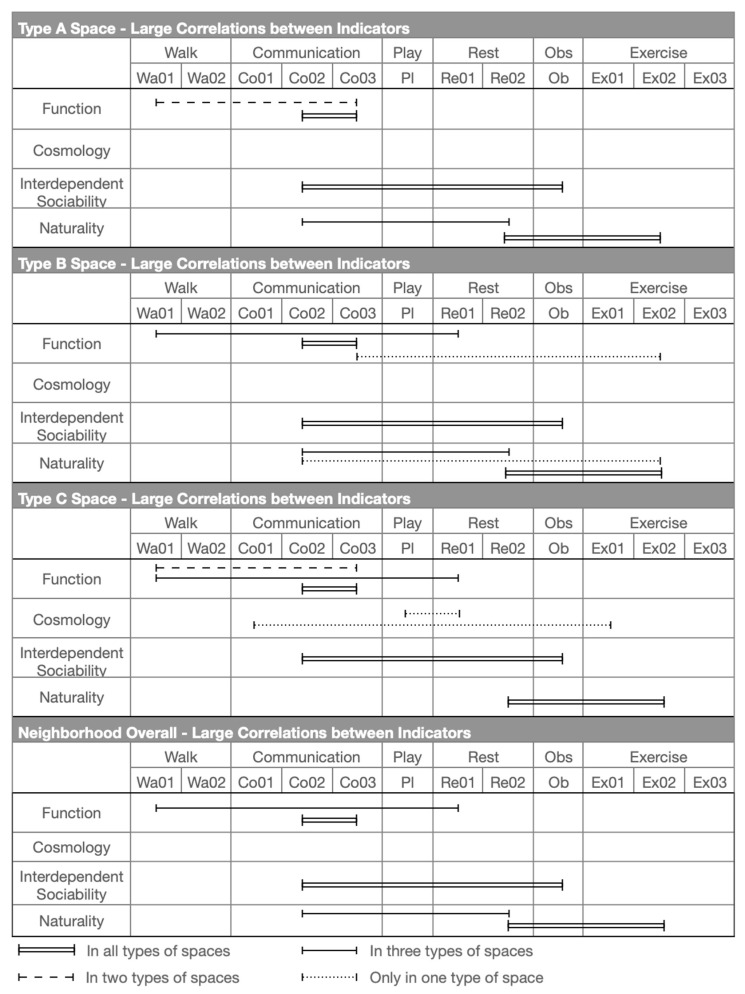
The large corrections between types of users’ behaviour in relation to local place qualities.

To evaluate users’ perceptions by applying local place qualities in a complex-developed neighbourhood, it is important to identify the socio-cultural responsiveness of the actual space to the adaption of local users’ perceptions and behavioural needs (Figure 10). It is also necessary to address users’ perceptions by applying local place qualities to find out which needs must be considered in design that can be defined as “good” for locals. Since the key aspects (health, enjoyment, and social dependence) are sometimes addressed by more than one local place quality, a correlation analysis is important for identifying how the quality or qualities affect users’ behavioural needs and in what specific ways, or how they interrelate to create combined effects.

Natural elements are highlighted in the use of all types of spaces in the neighbourhood, in which nature and humans are indivisible bodies (discussed in Section 3). As part of the natural procedure, human needs must be connected with natural design in both physical and mental terms to improve the state of human health [67,68]. From the physical side, this has become a basic requirement for improving people’s experiences during exercise and interactions with other people (Re02-Ex02). On the other hand, feeling connected to nature is mentally important, addressing both psychological satisfaction and spiritual achievement (Co02-Co03). This explains why local users enjoy social interaction and activities (Co02-Re02) (except in Type C Space) and enjoy watching interesting social actions and natural landscapes (Co02-Ob) while sitting in and engaging with the natural environment.

Another issue from cosmological cognitions in relation to function needs that have been addressed is the need to rest while walking in public spaces (except Type A Spaces) based on the philosophy of Chinese medicine (Wa01-Re01). As discussed in the walking section (Section 5.2), locals believe that walking is a type of exercise, an opportunity that allows users to integrate with nature and to improve their health [66,67]. Thus, resting facilities are strongly required while walking in order to enhance the exercise experience. Meanwhile, the sense of connection between the indoors and the outdoors is a significant factor that determines whether people choose walking as a way to access public spaces, and the frequency of use of these public spaces (Wa01-Co03).

Besides common findings applicable to all types of spaces, this study also identifies findings relevant to specific types of space. On the main roads of the community (Type B Space), local users strongly demand natural elements (greenery) in public spaces that could improve the experience of exercise (Co02-Ex02). In addition, the sense of connection between the indoors and the outdoors helps to determine the frequency of users exercising in this space (Co03-Ex02).

On the main streets around the community (Type C Space), active social interaction is associated with the natural environment because the quality of the natural setting is a significant factor that attracts local people to interact and behave in public open spaces (Co01-Ex01). In the meantime, local users prefer to rest and join social activities in these types of public spaces (Pl-Re01).

#### 6.3.2. Level of Impact of Local Place Qualities on Users’ Perception

In order to determine the level of impact of each indicator and their associated local place quality, Formula (4) is applied to calculate the score individually, which clearly indicates the level of impact for each indicator of local place qualities in all three types of spaces (alleyways, main roads, and main streets) and the overall neighbourhood.

**Figure 11:**
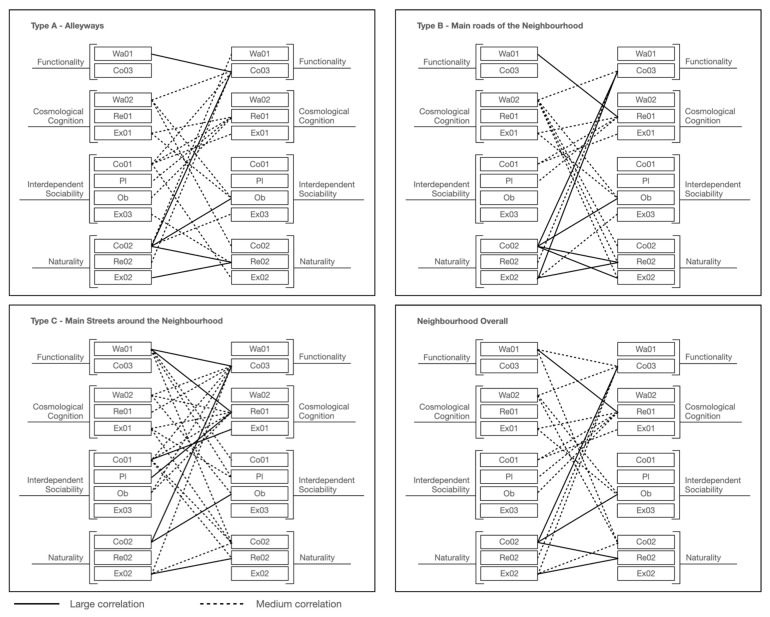
Medium and large correlations between indicators of local place qualities.

As shown in Figure 11, large and medium correlations (identified in Section 6.2, Table 10 and Table 11) between indicators of local place qualities are drawn to explore the number of interactional influences between each indicator of local place qualities in alleyways, main roads, main streets, and the neighbourhood as a whole.

**Figure 12:**
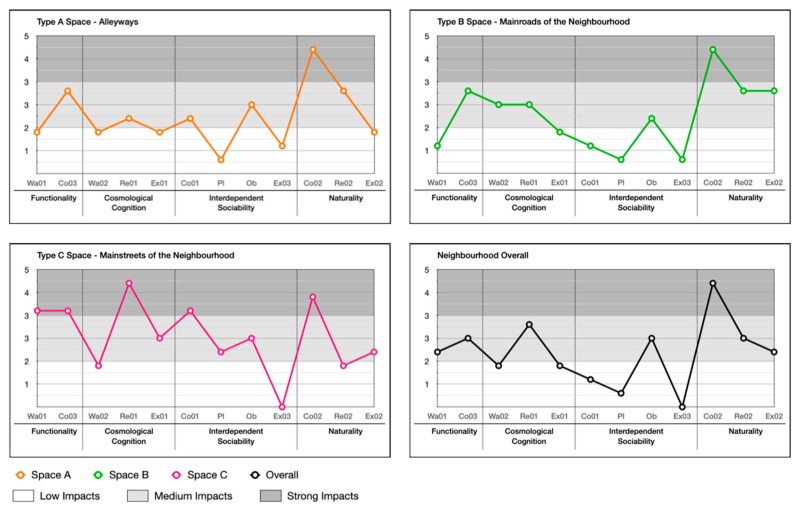
Impact score of each indicator of local place qualities on users’ perception of different types of space.

**Figure 13:**
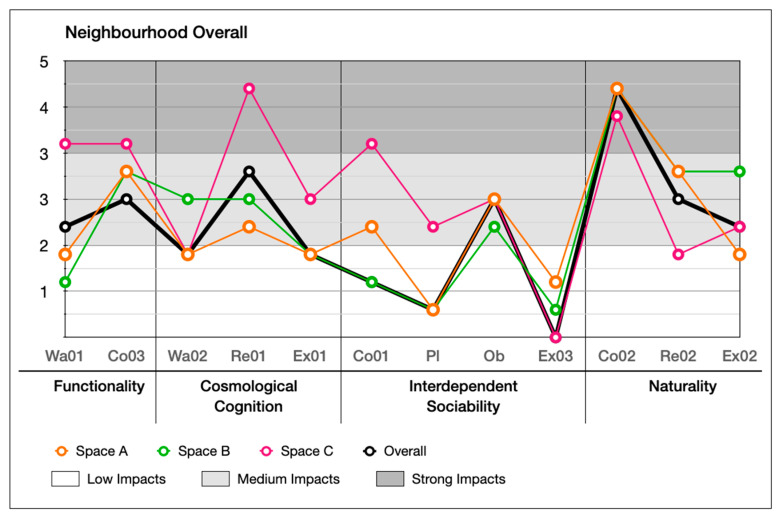
Comparison of impact score of indicators of local place qualities in different types of space.

**Figure 14:**
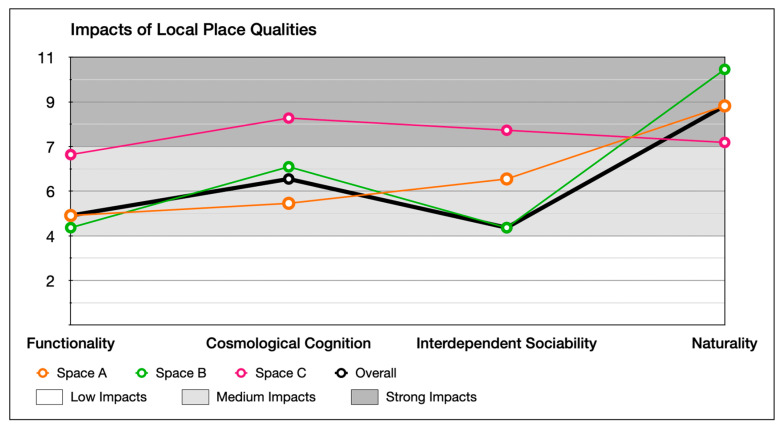
Comparison of impact score of local place qualities in different types of space.

**Figure 15:**
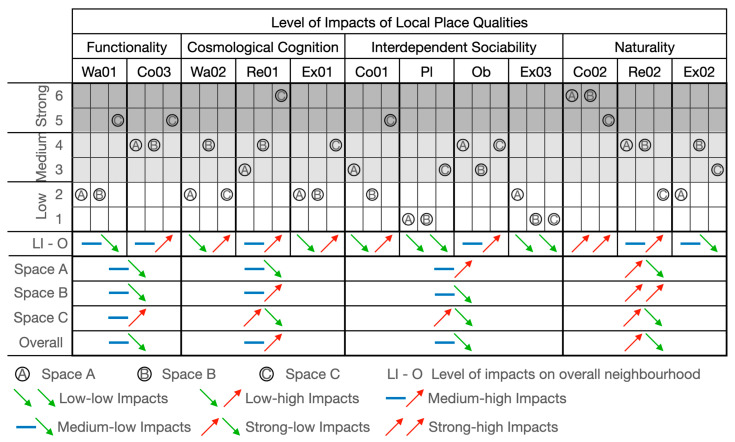
The level of impacts of local place qualities on users’ perceptions.

The calculation results are shown in Figure 12, Figure 13, Figure 14 and Figure 15. Naturality has an undeniable impact on users’ perceptions, particularly its indicator of Co02 (feeling related to natural elements) (refer to Table 4). We highlighted its significance across the alleyways, main roads, and main streets of the neighbourhoods. Interdependent sociability shows uncertain affection. Co01 (active interaction with others) addressed its importance from users’ perspectives, but Ex03 (group engagement activities) is presented as decreasing the impact on users’ perception among scales of space increase (from alleyways through main roads to main streets). Pl (ways of interacting with others) shows a contrast; when the scale decreases, its impact increases. Cosmological cognition is generally presented as having a medium-to-high impact on users’ perception. In particular, Re01 (temporary break) determines the importance of sitting facilities at all scales. Functionality shows great contrast in Wa01 (access for purpose) for the different types of spaces as local users demand variety in different scales of spaces.

## 7. Conclusions

This research aimed to identify local place qualities that could improve the adaptation and responsiveness to local users’ behavioural needs, as well as their perceptions of the contemporary urban setting. The results reveal that naturality is the most important quality of a location, together with functionality. This is related to the activities carried out at said location rather than cosmological cognitions, which have limited impact on the use of public spaces.

Naturality is the essence of local cognitions and the most important quality of a place, which directly frames local users’ individual behaviours to address health, enjoyment, and social dependence (such as the level of willingness of exercisers, and choices of a particular place for rest) and engagement in group activities (such as a particular place where social interactions act), indirectly creating the sense of connection between people and a place. Interdependent sociability has become a power factor, addressing local people’s interactional demands in types of spaces. However, the demand for group activity is decreasing with the changing scale of space from alleyways to main streets. On the other hand, the need for ways to interact with other people is increasing as the scale of space changes from main streets to alleyways. Surprisingly, cosmological cognitions show limited impacts compared to theories identified in the literature regarding the use of public spaces. This may be because the social ideology was transformed from hierarchical to collectivist in nature, and the original effects were largely replaced by social dependence.

On the other hand, health, enjoyment, and social dependence are highlighted as the most concerning factors, affecting the quality of use in public spaces that are addressed in the analysis. These factors are emphasized in different behavioural needs (walk, communication, play, rest, observation, and exercise) from the perspective of local cognitions. Particular meanings form in response to the behaviours and activities, creating unique patterns of uses. Influenced by naturality, interdependent sociability and cosmological cognitions, functionality has shown a level of impact on users’ perception at different scales of spaces, particularly in relation to walking demand, as it presents an opportunity for exercise, access to different areas, interactional opportunities, and observation of interesting actions.

The key contributions of the research are threefold: Firstly, local place qualities were systematically defined based on literature discussions and revised with on-site observations and interviews that can theoretically be traced back to remodify the local place qualities. Secondly, local understandings of public space were determined based on on-site investigations, which formed the first study defining the definition of public space based on the local Chinese perspective. Thirdly, the relevance between indicators of local place qualities was defined by analysing users’ perceptions of different types of public space. The results were used to refine the local place qualities and their impact on users’ perceptions. This can be seen as a set of tools used to evaluate whether the quality of a place meets local demands not only in Beijing, but also in the north of China, as the development of these cities is largely influenced by the experiences of Beijing’s residents, as well as its similar transformation stages and morphological settings.

The limitations of the study are as follows. Firstly, we must determine the local design qualities applicable in the south of China, which have yet to be tested, although these qualities are defined based on the key national-level theories. Secondly, the identified local design qualities were only tested in a complex-developed neighbourhood. The neighbourhood contains various historical, transitional, and modern building typologies that create diverse public spaces, but lack of analysis in the high-density neighbourhood case studies might lead to different results. Thirdly, selections of the random users as group in this case showed limitations, which resulted in two thirds of participants being female and over 50 years old. Although this is the representative situation in the case study area and more details were discussed in Section 5.2, more variable methods to identify perceptions from different age groups with relatively even numbers should be considered in further studies.

In order to reduce the limitations of the study, further research in the high-density neighbourhood is needed to identify how users’ perceptions are affected by local design choices in such a high-density modern built environment. Additionally, a case study in the south of China is planned to ascertain whether the aforementioned features are applicable. In addition, international cases may be involved in the further studies, which could allow identification of similarities and differences in these local place qualities that are tested in different contexts, especially in south-east Asia.

## Figures and Tables

**Table 1:** Changing of building types over time.

	Morphological Periods and Transforming of Buildings
Period 1	Imperial Period—before 1911
	Traditional housing types, open space structure and block layout, which address imperial power and social hierarchy in spatial structure.
Period 2	Soviet Influence Period—1949–1978
	Soviet socialist idea is spread into China, the housing types has changes to an early modern style to emphasize socialism and collectivism in lifestyle.
Period 3	Post-reform Period—1978–1990
	Modern building types with more private property layout design are imported into China, and the lifestyle are transformed into market lead.
Period 4	Contemporary—1990–present
	Not only modern building types developed, but also transitional building types become popular. Traditional culture is readdressed and historical buildings are proposed for protections and reuse under global influences.

**Table 2:** Frequencies for categorical demographic variables.

Personal Characteristics	*n*	Percent
Gender		
Female	119	66.11
Male	61	33.89
Total	180	100
Age		
22–35	38	21.11
35–50	12	6.67
>50	130	72.22
Total	180	100
Working Status		
Working	55	30.56
Studying	4	2.22
Not working	2	1.11
Retired	119	66.11
Total	180	100
Residential Characteristics	*n*	Percent
Role of status		
Residents	164	91.11
Work in the area	16	8.89
Total	180	100

**Table 3:** Key questions for defining behavioural needs between age groups.

Key Questions	Reasons	22–35 Years Old	35–50 Years Old	Above 50 Years Old	Percentage (%) of Total Participants
Why do people walk in public spaces	Access for destination	12	38	88	77
	Health behaviour	12	14	130	87
	I do not walk in public	0	0	0	0
Why do people communicate with other people	Social connection	12	28	109	83
	Social dependence	6	0	125	73
	I do not talk to others	0	2	0	1
Why do people exercise in public spaces	Self-training with no preferred space	12	5	14	17
	Green elements	0	0	72	40
	With other people	6	0	105	62
	I do not do exercise in public space	0	33	25	32
Why do people stop or break in public spaces	Temporary break	6	10	31	26
	For purposes, e.g., enjoy the sunshine, cool wind, or fresh air	6	0	68	41
	Talking to people	6	13	52	39
	Watching interests	0	6	10	9
	I do not stop or rest in public space	0	14	30	24
Why do people watch others in public spaces	Socially interesting	12	29	130	95
	Naturally interesting	6	23	82	62
	Building interesting	6	13	45	36
	I do not watch others	0	0	0	0
Why do people play in public spaces	Social attraction	6	0	120	70
	Limited indoor space	0	0	51	28
	I do not play in public space	0	33	10	24
	Natural attraction	6	5	0	6

**Table 4:** Labelling users’ behaviours with local cognitions beyond.

Users’ Behaviours	Label	Local Cognitions beyond the Behaviours
Walk	Wa01	Access for purposes
	Wa02	Health behaviour
Communication	Co01	Active interaction with others
	Co02	Feeling related to natural elements
	Co03	Sense of connection between indoor and outdoor
Play	Pl	Ways of interacting with others
Rest	Re01	Temporary break
	Re02	Customary relaxation
Observation	Ob	Visual interests
Exercise	Ex01	Exercising in the natural environment
	Ex02	Body training could be anywhere
	Ex03	Group engagement activities

**Table 5:** Local cognitions-based behaviours in relation to design qualities of spaces. The dark grey color indicates each local cognition based behaviour with relevant local place quality.

Local Place Qualities	Wa01	Wa02	Co01	Co02	Co03	Pl	Re01	Re02	Ob	Ex01	Ex02	Ex03
Naturality												
Cosmological Cognitions												
Interdependent Sociability												
Functionality												

**Table 6:** Interview questions for identifying local perceptions of the use of public spaces.

Local Place Qualities	Questions
Functionality	Wa01: How easy you feel to access the spaces?
	Co03: How you rate your feelings that access from inside to outside?
Cosmological Cognition	Wa02: How you rate your feelings to walk in the spaces as exercise or healthy activity?
	Re01: How you rate the spaces as a place for resting?
	Ex01: How you rate the spaces that feel enough space for your exercise needs
Interdependent Sociability	Co01: How you rate your feelings in communicating to other people (include vendors) in the spaces?
	Pl: How you rate the spaces as place for playing with others?
	Ob: How you rate the spaces as a place that have interesting things to watch?
	Ex03: How you rate the spaces as a place for doing exercise with other people?
Naturality	Co02: How you rate the spaces as good natural places to use or do activities?
	Re02: How you rate the spaces as a place to enjoy natural setting?
	Ex02: How you rate your feelings in doing exercise if there are enough natural plants?

Note: Items measured on a 5-point Likert scale ranging from “disagree” (1) to “agree” (5).

**Table 7:** Reliability test results of the research instrument based on datasets.

	Type A Space Data	Type B Space Data	Type C Space Data	General Neighbourhood Data
Cronbach’s Alpha	0.734	0.736	0.789	0.757
No. of Item	12	12	12	12

**Table 8:** Reliability test results of the research instrument based on local spatial qualities and indicators.

	Functions			Cosmological Cognition			Interdependent Sociability			Naturality		
	Wa01	Co03	Wa02	Re01	Ex01	Co01	Pl	Ob	Ex03	Co02	Re02	Ex02
Cronbach’s Alpha	0.762	0.750	0.766	0.759	0.773	0.783	0.794	0.759	0.788	0.724	0.754	0.765
No. of Item	12	12	12	12	12	12	12	12	12	12	12	12

**Table 9:** Validity test results of the research instrument based on datasets and indicators.

		Wa01	Co03	Wa02	Re01	Ex01	Co01	Pl	Ob	Ex03	Co02	Re02	Ex02
Type A Space	Pearson Correlation	0.315 ***	0.540 ***	0.474 ***	0.307 ***	0.289 ***	0.463 ***	0.582 ***	0.394 ***	0.411 ***	0.595 ***	0.539 ***	0.551 ***
	Sig. (2-tailed)	0.000	0.000	0.000	0.017	0.026	0.000	0.000	0.002	0.001	0.000	0.000	0.000
	*n*	60	60	60	60	60	60	60	60	60	60	60	60
Type B Space	Pearson Correlation	0.493 ***	0.394 ***	0.487 ***	0.498 ***	0.442 ***	0.310 ***	0.565 ***	0.535 ***	0.284 ***	0.532 ***	0.523 ***	0.339 ***
	Sig. (2-tailed)	0.023	0.002	0.000	0.000	0.000	0.016	0.000	0.000	0.028	0.000	0.000	0.008
	*n*	60	60	60	60	60	60	60	60	60	60	60	60
Type C Space	Pearson Correlation	0.434 ***	0.420 ***	0.481 ***	0.530 ***	0.312 ***	0.521 ***	0.386 ***	0.565 ***	0.496 ***	0.495 ***	0.351 ***	0.369 ***
	Sig. (2-tailed)	0.001	0.001	0.000	0.000	0.015	0.000	0.002	0.000	0.000	0.000	0.006	0.004
	*n*	60	60	60	60	60	60	60	60	60	60	60	60
General Neighbourhood	Pearson Correlation	0.508 ***	0.453 ***	0.259 ***	0.551 ***	0.374 ***	0.438 ***	0.498 ***	0.405 ***	0.146 ***	0.569 ***	0.326 ***	0.230 ***
	Sig. (2-tailed)	0.005	0.000	0.000	0.000	0.019	0.000	0.000	0.000	0.000	0.000	0.000	0.002
	*n*	180	180	180	180	180	180	180	180	180	180	180	180

*** present *p*-value less than 0.01.

**Table 10:** The correlation matrices for determining the relevance of indicators in the three types of spaces.

**Type A Space—Alleyways of the Community**												
	**Functionality**			**Cosmological Cognition**			**Interdependent Sociability**			**Naturality**		
	**Wa01**	**Co03**	**Wa02**	**Re01**	**Ex01**	**Co01**	**Pl**	**Ob**	**Ex03**	**Co02**	**Re02**	**Ex02**
**Wa01**	1.000	-	-	-	-	-	-	-	-	-	-	-
**Co03**	0.344 ***	1.000	-	-	-	-	-	-	-	-	-	-
**Wa02**	0.147	0.469 ***	1.000	-	-	-	-	-	-	-	-	-
**Re01**	0.509 ***	0.080	0.144	1.000	-	-	-	-	-	-	-	-
**Ex01**	0.133	−0.019	0.128	0.417 ***	1.000	-	-	-	-	-	-	-
**Co01**	0.186	−0.323 **	−0.069	0.410 ***	0.347 ***	1.000	-	-	-	-	-	-
**Pl**	0.246 *	0.030	0.045	0.434 **	0.227 *	0.188	1.000	-	-	-	-	-
**Ob**	0.245 *	0.268 **	0.419 ***	0.335 ***	0.408 ***	0.053	−0.164	1.000	-	-	-	-
**Ex03**	0.007	0.182	0.039	−0.056	0.025	−0.125	−0.079	0.064	1.000	-	-	-
**Co02**	0.331 ***	0.752 ***	0.467 ***	0.166	0.230 *	−0.187	−0.103	0.649 ***	0.302 **	1.000	-	-
**Re02**	0.145	0.432 ***	0.267 **	0.245*	0.125	0.044	−0.082	0.261 **	0.310 **	0.639 ***	1.000	-
**Ex02**	0.187	0.199	−0.068	0.098	−0.014	0.380 ***	0.019	−0.086	0.203	0.228 *	0.504 ***	1.000
**Type B Space—Main roads of the Community**												
	**Functionality**			**Cosmological Cognition**			**Interdependent Sociability**			**Naturality**		
	**Wa01**	**Co03**	**Wa02**	**Re01**	**Ex01**	**Co01**	**Pl**	**Ob**	**Ex03**	**Co02**	**Re02**	**Ex02**
**Wa01**	1.000	-	-	-	-	-	-	-	-	-	-	-
**Co03**	0.286 **	1.000	-	-	-	-	-	-	-	-	-	-
**Wa02**	0.064	0.445 ***	1.000	-	-	-	-	-	-	-	-	-
**Re01**	0.506 ***	0.079	0.145	1.000	-	-	-	-	-	-	-	-
**Ex01**	0.110	0.046	0.118	0.398 ***	1.000	-	-	-	-	-	-	-
**Co01**	0.258 **	−0.278 **	0.000	0.439 ***	0.308 **	1.000	-	-	-	-	-	-
**Pl**	0.228 *	0.038	−0.032	0.392 ***	0.151	0.061	1.000	-	-	-	-	-
**Ob**	0.081	0.157	0.455 ***	0.295 **	0.337 ***	0.158	−0.151	1.000	-	-	-	-
**Ex03**	−0.026	0.183	0.153	−0.150	−0.103	−0.075	−0.268 **	0.008	1.000	-	-	-
**Co02**	0.283 **	0.683 ***	0.481 ***	0.207	0.218 *	−0.094	−0.074	0.537 ***	0.181	1.000	-	-
**Re02**	0.131	0.453 ***	0.322 **	0.252 *	0.054	0.094	−0.125	0.215 *	0.283 **	0.725 ***	1.000	-
**Ex02**	0.048	0.520 ***	0.405 ***	0.068	−0.023	−0.094	−0.007	0.217 *	0.320 **	0.594 ***	0.732 ***	1.000
**Type C Space—Main streets around the Community**												
	**Functionality**			**Cosmological Cognition**			**Interdependent Sociability**			**Naturality**		
	**Wa01**	**Co03**	**Wa02**	**Re01**	**Ex01**	**Co01**	**Pl**	**Ob**	**Ex03**	**Co02**	**Re02**	**Ex02**
**Wa01**	1.000	-	-	-	-	-	-	-	-	-	-	-
**Co03**	0.648 ***	1.000	-	-	-	-	-	-	-	-	-	-
**Wa02**	0.294 **	0.410 ***	1.000	-	-	-	-	-	-	-	-	-
**Re01**	0.634 ***	0.391 ***	0.340 ***	1.000	-	-	-	-	-	-	-	-
**Ex01**	0.256 **	0.157	0.042	0.356 ***	1.000	-	-	-	-	-	-	-
**Co01**	0.362 ***	0.491 ***	0.225 *	0.433 ***	0.500 ***	1.000	-	-	-	-	-	-
**Pl**	0.346 ***	0.160	0.221 *	0.554 ***	0.337	0.194	1.000	-	-	-	-	-
**Ob**	0.290 **	0.320 **	0.467 ***	0.324 **	0.249 *	0.103	0.085	1.000	-	-	-	-
**Ex03**	−0.017	−0.036	−0.062	−0.111	−0.010	0.088	−0.092	0.031	1.000	-	-	-
**Co02**	0.464 ***	0.602 ***	0.287 **	0.267 **	0.316 **	0.344 ***	0.089	0.636 ***	0.019	1.000	-	-
**Re02**	0.163	0.265 **	−0.026	0.159	0.030	0.325 **	−0.072	0.147	0.004	0.549	1.000	-
**Ex02**	0.176	0.347 ***	0.077	0.084	−0.076	0.259 **	0.072	0.144	0.157	0.444 ***	0.571 ***	1.000

***, **, * present *p*-value less than 0.01, 0.05, 0.1. Small Correlation: r = 0.1; Medium Correlation: r = 0.3; Large Correlation: r = 0.5.

**Table 11:** The correlation matrix for determining the relevance of indicators on overall neighbourhood.

Overall Neighbourhood												
	Functionality			Cosmological Cognition			Interdependent Sociability			Naturality		
	Wa01	Co03	Wa02	Re01	Ex01	Co01	Pl	Ob	Ex03	Co02	Re02	Ex02
**Wa01**	1.000	-	-	-	-	-	-	-	-	-	-	-
**Co03**	0.398 ***	1.000	-	-	-	-	-	-	-	-	-	-
**Wa02**	0.166 **	0.451 ***	1.000	-	-	-	-	-	-	-	-	-
**Re01**	0.552 ***	0.164 **	0.210 ***	1.000	-	-	-	-	-	-	-	-
**Ex01**	0.166 **	0.057	0.109	0.394 ***	1.000	-	-	-	-	-	-	-
**Co01**	0.255 ***	−0.140 *	0.027	0.419 ***	0.365 ***	1.000	-	-	-	-	-	-
**Pl**	0.262 ***	0.058	0.055	0.442 ***	0.220 ***	0.135 *	1.000	-	-	-	-	-
**Ob**	0.211 ***	0.242 ***	0.444 ***	0.323 ***	0.338 ***	0.099	−0.092	1.000	-	-	-	-
**Ex03**	−0.018	0.121	0.049	−0.112	−0.034	−0.044	−0.160 **	0.020	1.000	-	-	-
**Co02**	0.360 ***	0.686 ***	0.422 ***	0.218 ***	0.255 ***	−0.022	−0.043	0.610 ***	0.160 **	1.000	-	-
**Re02**	0.140 *	0.400 ***	0.211 ***	0.218 ***	0.071	0.128 *	−0.099	0.203 ***	0.215 ***	0.639 ***	1.000	-
**Ex02**	0.129 *	0.353 ***	0.152 **	0.080	−0.038	0.154 **	0.020	0.099	0.232 ***	0.426 ***	0.614 ***	1.000

***, **, * present *p*-value less than 0.01, 0.05, 0.1. Small Correlation: r = 0.1; Medium Correlation: r = 0.3; Large Correlation: r = 0.5.

## Data Availability

The data presented in this study are available on request from the corresponding author. The data are not publicly available due to privacy concerns.
